# The association between oxidative balance score and periodontitis in adults: a population-based study

**DOI:** 10.3389/fnut.2023.1138488

**Published:** 2023-04-28

**Authors:** Haitao Qu

**Affiliations:** Department of Oral and Maxillofacial Surgery, Jinan Stomatological Hospital, Jinan, China

**Keywords:** Oxidative Balance Score (OBS), periodontitis, oxidative stress, NHANES, antioxidants

## Abstract

**Introduction:**

The pathogenesis between oxidative stress and periodontitis was correlated. The Oxidative Balance Score (OBS) is a systematic tool to assess the effects of diet and lifestyle in relation to oxidative stress. However, the association between OBS and periodontitis has not been reported previously.

**Methods:**

Sixteen dietary factors and four lifestyle factors were selected to score the OBS. Multivariate logistic regression and sensitivity analysis were used to investigate the relationship between OBS and periodontitis based on data from the National Health and Nutrition Examination Survey (NHANES) 1999–2018. Subgroup analysis and interaction tests were used to investigate whether this association was stable across populations.

**Results:**

This study included 3,706 participants. There was a negative linear association between OBS and periodontitis in all participants [0.89 (0.80, 0.97)], and after converting OBS to a quartile variable, participants with OBS in the highest quartile had a 29% lower risk of periodontitis than those with OBS in the lowest quartile [0.71 (0.42, 0.98)]. This negative association differed with respect to age and diabetes.

**Conclusion:**

There is a negative association between OBS and periodontitis in US adults. Our results suggest that OBS may be used as a biomarker for measuring periodontitis.

## 1. Introduction

Periodontitis is a common inflammatory disease of the oral cavity (1, 2), with the majority of cases occurring in people aged 55–59 years old (3), which is an important cause of tooth loss in adults (4). Plaque biofilm initiates the process, influencing the host’s immunological function and inflammatory response (5, 6). Bacterial and their metabolite-produced inflammatory mediators cause immunological dysfunction and periodontal tissue damage (7). Several previous epidemiological studies have demonstrated that periodontitis is a risk factor for various systemic illnesses (8, 9), with low-grade inflammation in the peripheral circulation being linked to the genesis and development of several diseases (10). Periodontitis has been linked to depression (11), Alzheimer’s disease (12), metabolic syndrome (13), and cardiovascular disease in various studies (14, 15).

An increasing body of research shows that the inflammatory response to periodontitis is linked to elevated local and systemic oxidative stress, as well as decreased antioxidant capacity (16, 17). Reactive oxygen species, which are primarily created in excess by hyperactive neutrophils as periodontitis develops, are not counterbalanced by the antioxidant defense system and cause tissue damage (18), increased metabolites of protein damage (19), DNA damage (20), and lipid peroxidation are characteristics of the process (21). Periodontitis may potentially have an impact on the antioxidants’ local and systemic actions (19).

The relationship between oxidative stress and periodontitis has attracted the interest of researchers. Over the past 40 years, studies have been reported on the association between several antioxidants and the prevalence of periodontitis (22–24). However, there are differences in data collection methods for dietary intake in many studies, which may explain the conflicting results of some studies (25). More importantly, antioxidants work systematically in concert, so measuring individual species in isolation can present limitations (26). For example, in addition to dietary factors, a number of lifestyle factors, including smoking (27), alcohol consumption (28), physical activity, and obesity (29, 30), also have an impact on organismal inflammation and oxidative stress.

The Oxidative Balance Score (OBS) is a composite indicator that assesses the oxidative balance of an individual (31). Generally, a higher OBS indicates a preference for antioxidants over pro-oxidants (32). The negative associations between OBS and a number of inflammation-related diseases has been found in several epidemiological studies, including cardiovascular disease (33), type 2 diabetes (34), chronic kidney disease (35), and osteoarthritis (36). However, no studies have assessed the association between OBS and periodontitis. Therefore, I conducted a cross-sectional study to examine the association between dietary and lifestyle integrated OBS and periodontitis according to the National Health and Nutrition Examination Survey (NHANES) 1999–2018.

## 2. Materials and methods

### 2.1. Study population

**FIGURE 1:**
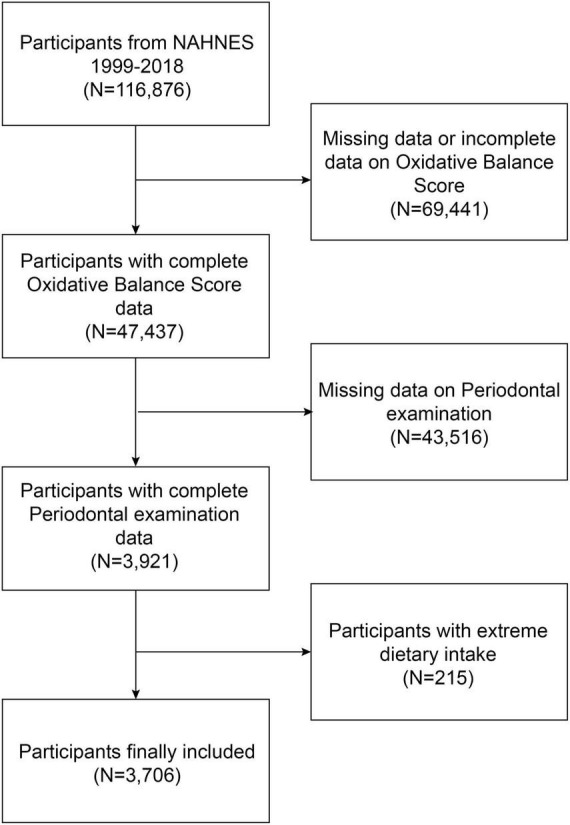
Flow chart of participants selection. NHANES, National Health and Nutrition Examination Survey.

The NHANES is a continuous nationwide survey that investigates the nutrition and health condition of adults and children in the United States (37, 38). The National Center for Health Statistics (NCHS) Research Ethics Review Board authorized the study protocol. At the time of recruiting, all participants provided written consent at the time of recruitment. This study utilizes the most recent five survey cycles of data from the last decade to conduct the survey. We excluded 69,441 participants without complete OBS data, 43,516 participants with missing periodontal examination data, and 215 extreme dietary intakes. The study eventually included 3,706 participants (Figure 1).

### 2.2. Oxidative Balance Score

Based on past research and experience, the OBS calculation combines the contributions of 16 dietary factors and 4 lifestyle factors, including 15 antioxidants and 5 pro-oxidants (39–42). Table 1 demonstrates the detailed scoring scheme of the OBS, with the first through third quartiles assigned a score of 0–2 for dietary antioxidants and 0 for pro-oxidants in the highest tertile and 2 in the lowest tertile. For lifestyle factors including physical activity (0 points for <400 MET-minute/week; 1 point for 400–1,000 MET-minute/week; 2 points for >1,000 MET-minute/week), alcohol intake (0 points for >30 g/d; 1 point for 0–30 g/d; 2 points for None), BMI (0 points for obesity. 1 for overweight; 2 for normal weight) and serum cotinine level (0 points for >0.038 ng/mL; 1 point for 0.038–1.13 ng/mL; 2 points for <1.13 ng/mL) (43, 44).

**TABLE 1:** Oxidative Balance Score assignment scheme.

OBS components	Property	Scoring assignment		
		**0**	**1**	**2**
**Dietary components**				
Dietary fiber (g/d)	Antioxidant	Tertile 1	Tertile 2	Tertile 3
Carotene (RE/d)	Antioxidant	Tertile 1	Tertile 2	Tertile 3
Riboflavin (mg/d)	Antioxidant	Tertile 1	Tertile 2	Tertile 3
Niacin (mg/d)	Antioxidant	Tertile 1	Tertile 2	Tertile 3
Vitamin B6 (mg/d)	Antioxidant	Tertile 1	Tertile 2	Tertile 3
Total folate (mcg/d)	Antioxidant	Tertile 1	Tertile 2	Tertile 3
Vitamin B12 (mcg/d)	Antioxidant	Tertile 1	Tertile 2	Tertile 3
Vitamin C (mg/d)	Antioxidant	Tertile 1	Tertile 2	Tertile 3
Vitamin E (ATE) (mg/d)	Antioxidant	Tertile 1	Tertile 2	Tertile 3
Calcium (mg/d)	Antioxidant	Tertile 1	Tertile 2	Tertile 3
Magnesium (mg/d)	Antioxidant	Tertile 1	Tertile 2	Tertile 3
Zinc (mg/d)	Antioxidant	Tertile 1	Tertile 2	Tertile 3
Copper (mg/d)	Antioxidant	Tertile 1	Tertile 2	Tertile 3
Selenium (mcg/d)	Antioxidant	Tertile 1	Tertile 2	Tertile 3
Total fat (g/d)	Prooxidant	Tertile 3	Tertile 2	Tertile 1
Iron (mg/d)	Prooxidant	Tertile 3	Tertile 2	Tertile 1
**Lifestyle components**				
Physical activity (MET-minute/week)	Antioxidant	<400	400–1,000	>1,000
Alcohol (g/d)	Prooxidant	>30	0–30	None
Body mass index (kg/m^2^)	Prooxidant	>30	25–30	<25
Cotinine (ng/mL)	Prooxidant	>0.038	0.038–1.13	<1.13

OBS, Oxidative Balance Score; RE, retinol equivalent; ATE, alpha-tocopherol equivalent; MET, metabolic equivalent.

### 2.3. Periodontitis

All participants in this study were examined by experienced dentists, and the specific examination procedures are described in the operating manual available on the NHANES website (45–47). For the classification of periodontal disease, we used the 2012 CDC/American Academy of Periodontology (AAP) case definition of periodontitis. Mild periodontitis was classified as two interproximal sites with attachment loss (AL) of three millimeters and two interproximal sites with pocket depth (PD) of four millimeters (not on the same tooth) or one site with PD of five millimeters. Moderate periodontitis was defined as two interproximal sites with AL 4 mm (not on the same tooth) or two interproximal sites with PD 5 mm (not on the same tooth). A total of 2 interproximal sites with AL 6 mm (not on the same tooth) and 1 interproximal site with PD 5 mm were categorized as severe periodontitis. The final number of periodontitis cases was the sum of mild, moderate, and severe cases (48).

### 2.4. Covariables

Covariates included age, gender, LDL-C (low-density lipoprotein cholesterol), race, diabetes, family income-to-poverty ratio (PIR), cancer, waist circumference, triglycerides, education level, and serum klotho levels. Detailed information on variable collection methods can be found in the NHANES Survey Methods and Analysis Guide.^1^

### 2.5. Statistical analysis

All analyses were performed with R (version 4.2) or Empowerstats (version 4.1). The chi-square test and *t*-test were used to assess the demographic characteristics of the participants by OBS quartile. Weighted multivariate logistic regression analyses were used to investigate the linear associations between OBS and periodontitis. After transforming OBS from a continuous variable to a categorical variable (quartile) a trend test was used to investigate the trend of linear association between OBS and periodontitis. Subgroup analysis was used to investigate the association between OBS and periodontitis in people of different gender, race, education, and diabetes status, and interaction tests were used to investigate whether the associations were consistent across subgroups. Statistical significance defined as two-sided *p* < 0.05.

## 3. Results

### 3.1. Baseline characteristics

At the time of assessment, the mean (SD) age of the 3,706 participants was 53.37 (14.89) years, 53.13% participants were females, and a total of 2,598 participants (70.08%) were diagnosed with periodontitis. In comparison to the bottom OBS quartile, participants in the top OBS quartile are more likely to be males, non-Hispanic white people and younger; In terms of socioeconomic status, higher OBS participants were more likely to have higher educational attainment and higher income; in terms of lifestyle, a lower proportion of higher OBS participants drank alcohol. In addition, participants in the lowest OBS quartile were more likely to with cancer and diabetes; to have lower BMI and waist circumference in terms of body size, and lower serum cotinine levels (Table 2). Supplementary Table 1 depicts the differences in clinical characteristics of participants with and without periodontitis.

**TABLE 2:** Basic characteristics of participants by Oxidative Balance Score quartile.

Characteristics	Oxidative Balance Score				*P*-value
	**Q1** ***N* = 927**	**Q2** ***N* = 926**	**Q3** ***N* = 926**	**Q4** ***N* = 927**	
Age (years)	54.80 ± 15.29	53.43 ± 14.49	52.97 ± 14.39	52.61 ± 14.24	0.018
Periodontitis, (%)					<0.001
Yes	75.13	70.58	63.44	52.67	
No	24.87	29.42	36.56	47.33	
Sex, (%)					<0.001
Male	36.57	47.08	55.83	62.89	
Female	63.43	52.92	44.17	37.11	
Race/Ethnicity, (%)					<0.001
Non-Hispanic white	47.03	48.16	51.51	54.37	
Non-Hispanic black	21.47	17.93	15.01	11.87	
Mexican American	15.53	17.49	19.33	19.20	
Other race/Multiracial	15.97	16.42	14.15	14.56	
Education level, *n* (%)					<0.001
Less than high school	37.22	30.67	27.11	22.33	
High school	25.89	21.60	23.33	18.55	
More than high school	36.89	47.73	49.56	59.12	
Smoking, (%)					0.206
Ever	50.05	46.44	47.30	45.31	
Never	49.95	53.56	52.70	54.69	
Drinking alcohol, (%)					0.006
Ever	71.81	61.12	62.35	58.37	
Never	28.19	38.88	37.65	41.63	
Cancer, (%)					0.341
Yes	10.79	9.83	9.18	12.51	
No	89.21	90.17	90.82	87.49	
Diabetes, (%)					0.001
Yes	15.43	15.77	10.04	10.25	
No	82.31	81.97	87.58	86.95	
Borderline	2.26	2.26	2.38	2.80	
BMI (kg/m^2^)	30.02 ± 6.99	29.98 ± 6.84	29.29 ± 6.21	28.69 ± 6.06	<0.001
Waist circumference (cm)	101.30 ± 15.91	101.69 ± 16.10	100.37 ± 14.98	98.99 ± 14.52	0.002
PIR	2.18 ± 1.51	2.46 ± 1.58	2.70 ± 1.63	3.00 ± 1.66	<0.001
Triglycerides (mg/dL)	141.84 ± 129.14	134.99 ± 106.87	139.92 ± 107.54	132.09 ± 143.62	0.514
Klotho (pg/mL)	813.23 ± 303.24	817.82 ± 277.08	831.34 ± 271.82	815.73 ± 313.40	0.823
LDL-C (mg/dL)	119.76 ± 34.45	120.93 ± 35.04	121.05 ± 35.01	116.19 ± 33.77	0.184
Serum cotinine (ng/ml)	98.92 ± 153.72	51.32 ± 122.73	36.25 ± 101.03	29.92 ± 95.40	<0.001

Mean ± SD for continuous variables: the *P*-value was calculated by the weighted linear regression model. (%) for categorical variables: the *P*-value was calculated by the weighted chi-square test. Q, quartile; PIR, ratio of family income to poverty; BMI, body mass index; LDL-C, low-density lipoprotein cholesterol.

### 3.2. Association between Oxidative Balance Score and periodontitis

Table 3 shows the association between OBS and periodontitis. We found higher OBS was negatively correlated with periodontitis both in crude model [0.79 (0.65, 0.92)] and adjusted model [0.82 (0.70, 0.93)]. After adjusted all covariables, each one-unit increase in the OBS score was found to be associated with an 11% decrease in the risk of periodontitis [0.89 (0.80, 0.97)]. After changing the OBS from a continuous to a categorical variable, sensitivity analyses were carried out. In the fully adjusted model, participants in the highest quartile had a 29% lower risk of periodontitis compared to those in the lowest quartile of OBS [0.71 (0.42, 0.98)]. Supplementary Tables 2, 3 demonstrate the association between OBS and periodontitis-related variables, with results showing that higher OBS scores are associated with lower C-reactive protein levels and with higher grades of self-reported oral health.

**TABLE 3:** The associations between Oxidative Balance Score and periodontitis.

Exposure	Model 1 [OR (95% CI)]	Model 2 [OR (95% CI)]	Model 3 [OR (95% CI)]
Oxidative Balance Score (continuous)	0.79 (0.65, 0.92)	0.82 (0.70, 0.93)	0.89 (0.80, 0.97)
**Oxidative Balance Score (quartile)**			
Quartile 1	reference	reference	reference
Quartile 2	0.91 (0.79, 1.03)	0.90 (0.81, 0.99)	0.93 (0.88, 0.98)
Quartile 3	0.83 (0.72, 0.94)	0.81 (0.72, 0.91)	0.84 (0.70, 1.00)
Quartile 4	0.53 (0.35, 0.72)	0.62 (0.39, 0.85)	0.71 (0.42, 0.98)
P for trend	<0.001	<0.001	<0.001

Model 1: No covariates were adjusted. Model 2: Age, gender, and race were adjusted. Model 3: Age, gender, race, diabetes, cancer, PIR, triglycerides, klotho, and LDL-C were adjusted. PIR, ratio of family income to poverty; LDL-C, low-density lipoprotein cholesterol.

### 3.3. Subgroup analyses

Subgroup analyses and interaction tests stratified by age, sex, race, BMI, and diabetes were performed to assess whether the relationship between OBS and periodontitis was consistent in the general population and to identify any potentially different population settings. Our findings showed that the associations were inconsistent. There was no statistical significance for gender, race, or BMI, as shown in Table 4, but we did find a significant interaction between age and diabetes (*P* for interaction <0.05). The negative association effect of OBS with periodontitis was significantly greater in older adults older than 60 years [0.83 (0.76, 0.91)] than in those younger than 60 years [0.91 (0.84, 0.98)]. In addition, this negative association effect was significantly greater in participants with diabetes [0.75 (0.56, 0.94)] than in those without diabetes [0.98 (0.95, 1.02)]. Although there were inconsistent effect values for the association between OBS and periodontitis in some subgroups, our results suggest that a negative association between OBS and periodontitis was maintained in all subgroups.

**TABLE 4:** Subgroup analysis of the association between Oxidative Balance Score and periodontitis.

Subgroup	Oxidative Balance Score [OR (95%CI)]	*P* for interaction
Sex		0.182
Male	0.85 (0.75, 0.95)	
Female	0.93 (0.90, 0.96)	
Age		0.046
<60 years	0.91 (0.84, 0.98)	
≥60 years	0.83 (0.76, 0.91)	
Race/Ethnicity		0.089
Non-Hispanic white	0.88 (0.81, 0.95)	
Non-Hispanic black	0.79 (0.63, 0.95)	
Mexican American	0.94 (0.85, 1.03)	
Other race/Multiracial	0.92 (0.89, 0.96)	
Education level, *n* (%)		0.579
Less than high school	0.85 (0.72, 0.98)	
High school	0.91 (0.82, 1.00)	
More than high school	0.81 (0.70, 0.92)	
Diabetes, (%)		0.028
Yes	0.75 (0.56, 0.94)	
No	0.98 (0.95, 1.02)	
Borderline	0.93 (0.91, 0.95)	

Age, gender, race, diabetes, cancer, PIR, triglycerides, klotho, and LDL-C were adjusted. PIR, ratio of family income to poverty; LDL-C, low-density lipoprotein cholesterol.

## 4. Discussion

In the cross-sectional study that enrolled 3,706 representative participants, we observed a negative association between the OBS and periodontitis, and there was significant dependence of age and diabetes on this association, indicating that an increased OBS may contribute to a decreased risk of periodontitis. Our results suggest that the management of OBS in dietary intake and lifestyle may alleviate the occurrence of periodontitis.

To our knowledge, this is the first study to assess the relationship between OBS and periodontitis, and it highlights the negative association between OBS levels derived from dietary intake and lifestyle and the risk of periodontitis. Previous studies have found that oxidative stress has a negative impact on periodontitis risk and oral health (49). Tamaki et al. (50) investigated the association between serum oxidative stress levels and periodontitis in 200 adult participants from the community with periodontitis. The results of the age-adjusted logistic analysis showed a statistically significant association between high ROM levels and clinical attachment loss (50). In a cohort study that included 770 participants with chronic kidney disease (CKD), Sharma et al. (51) attempted to investigate the causal association between oxidative stress, periodontitis, and renal function using a mediator analysis model, and the authors found a bidirectional negative association between periodontitis and renal function, with oxidative stress providing the pathobiological basis for this relationship. Li et al. investigated the association between four serum antioxidant vitamins (vitamins A, C, D, and E) and periodontitis in a cross-sectional study that included 6,158 Americans. The results showed a significant negative association between vitamins C and D and periodontitis, and in addition, the authors concluded that periodontitis increased the level of systemic inflammation in the obese population. In our analysis, we detected a linear negative association between OBS and periodontitis (48). A trend test considering OBS as a quartile also demonstrated a dose-response relationship between OBS and periodontitis. It has been widely reported that OBS can be used as an indicator of inflammatory diseases (52), and the association between OBS and periodontitis was also recognized in our study.

The results of the subgroup analysis showed significant differences in the association between OBS and periodontitis with respect to age and diabetes, which is partially consistent with previous studies. Ebersole et al. (53) evaluated the association between five antioxidants (folate, vitamin D, vitamin E, *cis*-beta-carotene, and β-cryptoxanthin). The authors found an interaction between age and periodontitis-related levels of these nutrients, with reduced levels of these antioxidants increasing with age in moderate and severe periodontitis (53). Our interaction test showed that the negative association effect between OBS and periodontitis was more significant in the elderly. Furthermore, diabetes and periodontitis share a common pathogenesis associated with altered immune inflammatory responses at the systemic level (54). An animal study showed that periodontitis exacerbates oxidative stress levels in rats with diabetes (55). Our results also suggest that participants with diabetes are more prominent in the negative association between OBS and periodontitis.

The role of oxidative stress and inflammation in the pathogenesis of periodontitis has attracted the attention of researchers for decades (56, 57). Although many past observational cross-sectional studies have confirmed the association of oxidative stress with periodontitis, the low specificity of oxidative stress markers requires caution in interpreting the results, and a meta-analysis that included 16 observational studies outlined the substantial heterogeneity introduced by differences in patient populations and analytical tools (58). In fact, with the exception of vitamin C, which is considered a well-known strong antioxidant, the associations between other dietary components and inflammatory diseases are often conflicting (59, 60). Therefore, the introduction of a comprehensive scoring system reflecting dietary and non-dietary antioxidant and pro-oxidant exposures to assess the relationship between oxidative stress and periodontitis is warranted (61). OBS, which combines dietary and lifestyle factors, has been shown to be a useful marker for inflammatory diseases in studies in different countries and regions (43, 62, 63).

The strengths of our study include the simultaneous consideration of multiple dietary and lifestyle factors for the oxidative potential of periodontitis; secondly, the use of a complex multi-stage probability sampling design and appropriate covariate adjustment increased the reliability and representativeness of our study. Our study has some limitations. First, due to the design of the cross-sectional study, a causal relationship between OBS and periodontitis could not be inferred (64). In addition, database limitations prevented the inclusion of all covariates that have an impact on oxidative stress, such as environmental pollution, flavonoid intake, and Oxidative markers (65). Nevertheless, the correlation between periodontitis and current OBS was stable enough to be less likely to be significantly influenced by unincluded factors.

## 5. Conclusion

In conclusion, higher OBS indicates that dietary and lifestyle antioxidant exposure is superior to prooxidant exposure and is associated with a lower risk of periodontitis. Our results suggest that OBS may serve as a biomarker for periodontitis in adults. However, further studies are still needed to validate our findings.

## Data availability statement

Publicly available datasets were analyzed in this study. This data can be found here: www.cdc.gov/nchs/nhanes/.

## Ethics statement

The studies involving human participants were reviewed and approved by the National Center for Health Statistics (NCHS) Research Ethics Review Board. The patients/participants provided their written informed consent to participate in this study.

## Author contributions

HQ read and approved the final manuscript, performed the analysis, wrote a draft of this manuscript, conceived the study design, contributed to the interpretation of the results, and critically revised the manuscript for important intellectual content.

## Footnotes

1
https://wwwn.cdc.gov/nchs/nhanes/analyticguidelines.aspx
