# Supplementary material for: The association between oxidative balance score and periodontitis in adults: a population-based study
Source: Front Nutr. 2023 Apr 28;10:1138488. doi: 10.3389/fnut.2023.1138488 (PMC10178495; doi:10.3389/fnut.2023.1138488)
Supplement: Supplementary file 1 [file Table_1.DOCX]

**Table S1:** Basic characteristics of participants by periodontitis status among.

| **Characteristics** | **Periodontitis**  **(2392)** | **Non-periodontitis (1314)** | ***P*-value** |
| --- | --- | --- | --- |
| Age (years) | 55.51±16.62 | 52.01±15.58 | <0.001 |
| Self-reported Oral health, (%) |  |  | <0.001 |
| Excellent | 10.9 | 16.8 |  |
| Very good | 21.9 | 26.6 |  |
| Good | 35.2 | 34.8 |  |
| Fair | 19.7 | 14.5 |  |
| Poor | 12.3 | 7.3 |  |
| Sex, (%) |  |  | <0.001 |
| Male | 47.18 | 53.07 |  |
| Female | 52.82 | 46.93 |  |
| Race/ethnicity, (%) |  |  | <0.001 |
| Non-Hispanic White | 35.52 | 34.80 |  |
| Non-Hispanic Black | 34.57 | 24.18 |  |
| Mexican American | 11.97 | 13.75 |  |
| Other race/multiracial | 17.94 | 27.27 |  |
| Education level, n (%) |  |  | <0.001 |
| Less than high school | 27.92 | 17.98 |  |
| High school | 24.88 | 21.99 |  |
| More than high school | 47.20 | 60.03 |  |
| Smoking, (%) |  |  | <0.001 |
| Ever | 61.41 | 47.10 |  |
| Never | 38.59 | 52.90 |  |
| Drinking alcohol, (%) |  |  | <0.001 |
| Ever | 69.71 | 68.00 |  |
| Never | 30.29 | 32.00 |  |
| Cancer, (%) |  |  | <0.001 |
| Yes | 10.23 | 9.38 |  |
| No | 89.77 | 90.62 |  |
| Diabetes, (%) |  |  | <0.001 |
| Yes | 15.11 | 10.32 |  |
| No | 82.53 | 87.57 |  |
| Borderline | 2.36 | 2.11 |  |
| BMI (kg/m^2^) | 30.62 | 28.51 | <0.001 |
| Waist circumference (cm) | 100.89 ± 15.33 | 98.79 ± 13.50 | <0.001 |
| PIR | 2.36 ± 1.67 | 2.71 ± 1.80 | <0.001 |
| Triglycerides (mg./dL)  Klotho (pg/mL)  LDL-C (mg/dL) | 137.64± 107.59  815.14 ± 257.89  118.63 ± 41.51 | 135.91 ± 103.18  820.14 ± 281.02  115.40 ± 36.79 | 0.281  0.051  0.193 |
| Serum cotinine (ng/ml) | 76.02 ± 125.75 | 34.26 ± 89.01 | <0.001 |
| C-Reactive Protein (mg/L) | 4.34 ± 2.69 | 2.79 ± 2.03 | <0.001 |

Mean ± SD for continuous variables: the P value was calculated by the weighted linear regression model.

(%) for categorical variables: the P value was calculated by the weighted chi-square test.

Abbreviation: Q, quartile; PIR, Ratio of family income to poverty; BMI, body mass index; LDL-C, low-density lipoprotein cholesterol.
