# Supplementary material for: The association between oxidative balance score and periodontitis in adults: a population-based study
Source: Front Nutr. 2023 Apr 28;10:1138488. doi: 10.3389/fnut.2023.1138488 (PMC10178495; doi:10.3389/fnut.2023.1138488)
Supplement: Supplementary file 2 [file Table_2.DOCX]

**Table S2:** The associations between Oxidative Balance Score and C-Reactive Protein.

| **Exposure** | **Model 1 [β (95% CI)]** | **Model 2 [β (95% CI)]** | **Model 3 [β (95% CI)]** |
| --- | --- | --- | --- |
| Oxidative Balance Score (continuous) | -1.05 (-1.56, -0.05) | -0.78 (-1.06, -0.10) | -0.49 (-0.72, -0.27) |
| Oxidative Balance Score (quartile) |  |  |  |
| Quartile 1 | reference | reference | reference |
| Quartile 2 | -0.41 (-1.13, -0.46) | -0.35 (-0.82, -0.21) | -0.28 (-0.32, -0.23) |
| Quartile 3 | -0.94 (-2.32, -0.43) | -0.62 (-1.25, 0.31) | -0.51 (-0.68, -0.34) |
| Quartile 4 | -1.26 (-2.94, -0.22) | -0.88 (-1.62, -0.15) | -0.17 (-0.75, -0.08) |
| P for trend | < 0.001 | < 0.001 | < 0.001 |

Model 1: no covariates were adjusted. Model 2: age, gender, and race were adjusted. Model 3: age, gender, race, diabetes, cancer, PIR, triglycerides, klotho, and LDL-C were adjusted. Abbreviation: PIR, Ratio of family income to poverty; LDL-C, low-density lipoprotein cholesterol.
