# Supplementary material for: The association between oxidative balance score and periodontitis in adults: a population-based study
Source: Front Nutr. 2023 Apr 28;10:1138488. doi: 10.3389/fnut.2023.1138488 (PMC10178495; doi:10.3389/fnut.2023.1138488)
Supplement: Supplementary file 3 [file Table_3.DOCX]

**Table S3:** Negative binomial regression model showing the associations between Oxidative Balance Score and Self-reported Oral health.

| **Exposure** | **Fully adjusted model [RR (95% CI)]** |
| --- | --- |
| **Oxidative Balance Score** |  |
| Excellent | reference |
| Very good | 0.96 (0.92, 0.99) |
| Good | 0.87 (0.81, 0.93) |
| Fair | 0.63 (0.48, 0.78) |
| Poor | 0.55 (0.31, 0.70) |

Age, gender, race, diabetes, cancer, PIR, triglycerides, klotho, and LDL-C were adjusted. Abbreviation: PIR, Ratio of family income to poverty; LDL-C, low-density lipoprotein cholesterol.
